# The International Immunotherapy Society for Fungal Diseases

**DOI:** 10.1128/aac.01774-25

**Published:** 2026-02-04

**Authors:** Arturo Casadevall, Dimitrios P. Kontoyiannis, Frank L. van de Veerdonk

**Affiliations:** 1 Department of Molecular Microbiology and Immunology, Johns Hopkins School of Public Health 25802 , Baltimore, Maryland, USA; 2 Division of Internal Medicine, The University of Texas MD Anderson Cancer Center, Houston, Texas, USA; 3 Department of Internal Medicine and Radboud Center for Infectious Diseases, Radboud University Medical Center 6034, Nijmegen, the Netherlands

**Keywords:** fungal, immunocompromised, immunotherapy

## Abstract

The treatment of invasive fungal diseases is unsatisfactory because of high morbidity and mortality despite antifungal therapy. These patients are often immunocompromised, and improvements in treatment outcome are likely to require immune therapy. To promote immune therapies against fungal diseases, the International Immunotherapy Society for Fungal Diseases was organized.

## EDITORIAL

In the spring of 2024, the National Institutes of Health hosted a 2-day meeting in Rockville, Maryland, USA, titled “Realizing the promise of adjunctive immune therapy for invasive fungal infections” that was organized by Drs. Donna Love and Baoying Liu. The goal was to bring together pre-clinical and clinical investigators working on immunotherapies for treating fungal diseases. The conference highlighted the fact that while there was great interest in immunotherapy for fungal diseases, and there were many promising projects, most investigators and their projects were isolated efforts that could benefit from coalescing into a field. During a coffee break at the conference, your authors discussed the need for an organization that would promote immunotherapy for fungal diseases and thus the germ for a possible The International Immunotherapy Society for Fungal Diseases (IISFD) was planted. In the year following the conference, your authors met regularly and discussed the idea of a new society with colleagues at their institutions and conferences, and the plan began to gel. In the summer of 2025, we reached out to leaders in the field, and the new society was officially formed with the formation of a 13-member board of directors (listed in https://iisfd.org/) who held their inaugural meeting in September 2025. The inaugural conference for the IISFD is being planned for summer 2026.

The promise of immunotherapy for fungal diseases is in sync with the tenor of our times. In the third decade of the 21st century, immune therapies have revolutionized the treatment of many diseases such as cancer, but this revolution has largely bypassed the field of infectious diseases, which remains highly focused on promoting the use and development of anti-infectives. The stagnation of immune therapies for infectious diseases is paradoxical, given that this field developed serum therapy, which was the first effective immunotherapy in the early 20th century to treat a variety of bacterial and viral diseases (1). The causes for the underdevelopment of immune therapies for the treatment of infectious diseases are complex and include the existence of antimicrobial drugs for many conditions, small markets for specific therapies, the dynamic and heterogeneous evolution of immune status in the setting of a fungal infection, and a preference for non-specific therapies capable of treating many microbial diseases (2).

Today, there is a great need for new therapeutic approaches in the management of invasive fungal diseases, which carry high morbidity and mortality even when treated only with antifungal drugs. Although there has been consistent progress in the management of fungal diseases, this progress has been incremental, and there is a need for reassessing our approach to antifungal therapy as we may have reached the “ceiling effect” of the maximum efficacy of modern antifungals in fungal diseases. For example, cryptococcosis was uniformly fatal until the introduction of polyene amphotericin B in the late 1950s when it became possible to save about half of affected individuals, and while its prognosis has improved steadily over subsequent decades, the mortality remains somewhere between 10% and 20%, which is unacceptably high, and many survivors are left with debilitating long-term sequelae. The overwhelming majority of invasive fungal diseases occur in hosts with impaired immunity, which can result from innate errors of metabolism, infection with immunosuppressive viruses such as HIV, and the use of immunomodulating therapies that suppress the immune system in the treatment of malignancy, autoimmune processes, or transplantation. In such immunocompromised hosts, antifungal therapy does not work fully given the need for immune function to eradicate infection (3). Hence, it may be possible to improve therapeutic outcomes by providing therapy that enhances residual immunity and/or recruits other aspects of the immune system to fight the infection. An example of this principle is apparent in the outcome of cryptococcosis in patients with acquired immunodeficiency syndrome, which was incurable in the setting of the severe HIV-associated immune deficits and required lifelong maintenance therapy but can now be cured when the immune system rebounds with antiretroviral therapy. However, that outcome can be complicated by the immune reconstitution inflammatory syndrome (IRIS), highlighting the double-edged nature of immunity in infectious disease. Also, the outcome of most opportunistic infections in oncology is mainly host-driven (4). We believe that for those very difficult-to-treat invasive fungal diseases such as mucormycosis, disseminated coccidioidomycosis, aspergillosis, etc., it will be possible to improve their prognosis by augmenting or inducing host immunity (5), including cell therapies, immunomodulating small molecule delivery, and next-generation vaccine development with implantable biomaterials.

Today, the biomedical revolution that began in the 1950s has generated a plethora of new therapeutic options that include monoclonal antibodies, cytokines, small molecule immunomodulators, engineered T cells, etc. that could potentially be used as adjunctive therapy for invasive fungal diseases. Unfortunately, beyond empiricism, we do not know how to take advantage of this arsenal because the necessary pre-clinical and clinical studies have not been completed, and our understanding of antifungal immune responses in health and disease has been fragmented. There are many impediments to learning how to use immunotherapy for fungal diseases as there are many challenges in the feasibility of pragmatic clinical immunotherapy trials that include inadequate funding, heterogeneity and rarity of fungal diseases, and lack of leadership in championing immune-based therapies. In addition, immune interventions based on profiling of immune dysfunction in fungal infection are lacking, although some successful attempts using translational concepts are emerging (6). Hence, we have formed the IISFD to become an organized proponent of immune therapies for fungal diseases. In 2025, the IISFD was incorporated as a non-profit organization with the following goals:

1. **Advance Immunotherapy**. IISFD will foster exploration, development, and application of innovative immunotherapeutic strategies aimed at improving the treatment and management across the spectrum of fungal infections, with an overarching goal of enhancing patient health outcomes.

2. **Promote Host-Directed Interventions**. The IISFD will support the research and advancement of host-directed therapies that aim to optimize the body’s immune response, thereby improving the management and prevention of fungal infections and related diseases.

3. **Encourage Knowledge Sharing and Education**. The IISFD will promote the dissemination of knowledge and facilitate education on the role of host-pathogen interactions in the context of fungal infections. This includes encouraging research, collaboration, and the exchange of best practices to foster a deeper understanding of immune-based therapeutic strategies.

4. **Support Collaborative Research and Development**. The IISFD will encourage and support collaborative efforts across academic, clinical, industry, and government sectors to advance the scientific understanding and clinical application of immunotherapy for fungal diseases.

Looking ahead, the IISFD envisions that education in fungal immunotherapy will shift from passive knowledge transfer to competency-based training that equips clinicians to interpret the status of the immune function and act on it. To support this, we will establish an international fungal immunotherapy academy that trains a new generation of clinician-scientists fluent in artificial intelligence, systems immunology, and innovative trial design. In parallel, cell-based therapies targeting fungi, engineered T cells, natural killer cells, and macrophages will evolve from experimental concepts into scalable, off-the-shelf treatments, designed and optimized through artificial intelligence (AI)-guided discovery and high-throughput screening to recognize fungal patterns. We envisage that immunotherapy trials will become routine, embedded in a global platform where adaptive protocols learn from each individual patient in real time and single-patient insights are systematically aggregated into new standards of care. By integrating multi-omics, clinical data, imaging, and even AI-driven “digital mirrors” of the immune system will allow us to simulate and refine fungal immunotherapies before they are given at the bedside.

We are aware that the successful implementation of immunotherapy will require a collaborative effort that will include basic scientists, treating physicians, educational programs, pharmaceutical companies, regulatory bodies, and funding agencies. Immunotherapy includes a set of interventions that range from modulating the immune response to reducing inflammatory damage to enhancing the antimicrobial efficacy of immune effector cells. We envision that IISFD will become a big tent that could help bring together these different groups with the overarching goal of improving outcomes for those who suffer from fungal diseases. For this approach to succeed, physicians will have to learn whether the patients need up- or downregulation of the immune response and how to evaluate in real time that dynamic balance in the context of the natural history of a fungal infection and its treatment. A helpful qualitative approach is provided by the damage-response of microbial pathogenesis, which denotes the host-microbe interaction as a parabola where each affected patient can be located by strength of their immune response and the damage resulting from the host-microbe interaction, which can provide an “immune vector.” Importantly, their position on the parabola, or this immune vector, is amenable to movement by the institution of immunotherapy. Continuing with the cryptococcal examples mentioned above, patients with insufficient immune response, such as those with advanced HIV infection, may benefit from immune-enhancing interventions, such as the addition of gamma interferon (7), while more immunologically intact individuals who mount strong inflammatory responses (8) or those experiencing IRIS after initiation of antiretroviral therapy may benefit from immunosuppressive interventions (9).

The IISFD will be a scientific society, and such societies have been defined as “an association of people who come together to promote progress in a specific technological or scientific area and to facilitate the interaction of interested people on a regional, national, or international level” (10). The IISFD seeks to promote immunotherapy along the entire spectrum from basic science to clinical applications and foster cross-fertilization between disciplines directly or indirectly related to fungal immunity. Today, while we are focusing on fungal diseases, we are fully aware that immune therapies could also improve the outcome of other infectious diseases such as those caused by viruses, bacteria, mycobacteria, and parasites. We are intentionally focusing initially only on fungal diseases because there is a major unmet need due to poor response to antifungals in the setting of impaired immunity. By beginning with a narrow focus on fungal diseases, we hope to learn how to promote immunotherapy for a group of diseases where there is a great need for new approaches. We are hopeful that the IISFD will succeed in promoting immunotherapy and anticipate a day when this society’s name evolves into IISID, which will be the International Immunotherapy Society for Infectious Diseases.
